# Seroprevalence of melioidosis and its association with blood profiles and pathogens in sheltered dogs in southern Thailand

**DOI:** 10.14202/vetworld.2024.705-711

**Published:** 2024-03-25

**Authors:** Punpichaya Fungwithaya, Worakan Boonhoh, Narin Sontigun, Orachun Hayakijkosol, Wiyada Kwanhian Klangbud, Tuempong Wongtawan

**Affiliations:** 1 Office of Administrative Interdisciplinary Program on Agricultural Technology, School of Agricultural Technology, King Mongkut’s Institute of Technology Ladkrabang, Bangkok 10520 Thailand; 2 Akkhraratchakumari Veterinary College, Walailak University, Nakhon Si Thammarat 80160, Thailand; 3 Centre for One Health, Walailak University, Nakhon Si Thammarat 80160, Thailand; 4 Division of Tropical Health and Medicine, College of Public Health, Medical and Veterinary Sciences, James Cook University, Queensland, Australia; 5 Centre of Excellence Research for Melioidosis and Microorganisms, Walailak University, Nakhon Si Thammarat 80160, Thailand; 6 Department of Medical Technology, School of Allied Health, Sciences, Walailak University, Nakhon Si Thammarat 80161, Thailand

**Keywords:** blood pathogens, dogs, hematology, melioidosis, prevalence, serum biochemistry

## Abstract

**Background and Aims:**

Melioidosis is a notable zoonotic disease in Thailand that can affect both humans and animals. Although dogs are one of the most popular pets worldwide, there is a remarkable lack of information on the prevalence and knowledge of canine melioidosis. This study aimed to estimate the seroprevalence of melioidosis in sheltered dogs and its relationship with the blood profile and blood pathogens.

**Materials and Methods:**

Melioidosis in 156 dogs was analyzed using an indirect hemagglutination assay. Hematology and serum biochemistry tests were performed using an automated system. Blood pathogens (e.g., *Ehrlichia*, *Anaplasma*, *Hepatozoon*, and *Babesia*) were diagnosed using conventional polymerase chain reaction.

**Results:**

The seroprevalence rates of canine melioidosis and blood pathogen infection were 5.77% (9/156) and 50.64% (79/156), respectively. Seropositive dogs generally have higher lymphocyte counts and aspartate aminotransferase levels but lower total white blood cell, neutrophil, and platelet (PLT) counts than seronegative dogs. No statistically significant difference (p > 0.05) was observed between the seropositive and seronegative dogs’ hematology and serum biochemistry findings. Neither the correlation between melioidosis and blood pathogen infection nor the association between melioidosis and thrombocytopenia was statistically significant (p > 0.05). Remarkably, dogs that had coinfections with both melioidosis and blood pathogens demonstrated a significantly reduced PLTcount (49,167 ± 7,167) compared with dogs that tested positive for melioidosis but negative for blood pathogens (139,333 ± 29,913) (p < 0.01).

**Conclusion:**

In southern Thailand, the prevalence of canine melioidosis was low but the prevalence of blood pathogens was high. Coinfection with blood pathogens can significantly reduce PLT counts, which may have a potentially serious impact. Future research should focus on conducting seroprevalence studies in the general dog population.

## Introduction

As per the estimation, 700 million dogs exist worldwide, 70% of which are stray and roaming animals [1]. In Thailand, stray and free-ranging dogs account for more than 50% of the total dog population [2]. Stray dogs can cause road accidents and harm human health by attacking, biting, and spreading rabies, leptospirosis, parasitic disease, and melioidosis to other animals and humans [2–7]. Shelters are used to help control the population of stray and free-ranging dogs in our community. Shelters, however, can be a source of numerous diseases because they often contain several dogs and have different management styles. Only a few studies have been conducted on the health and illness of sheltered dogs, especially in developing countries. In Thailand, 70.1% of sheltered dogs carry zoonotic intestinal antimicrobial bacteria and approximately 40% carry helminths and protozoa [8–11].

Melioidosis caused by *Burkholderia pseudomallei* is an important zoonotic bacterial disease in Thailand and is commonly found in soil and water [12, 13]. Melioidosis can cause death in humans as well as in a wide variety of animals, including goats, pigs, cattle, deer, camels, crocodiles, zebras, and monkeys [13]. The mortality rate in humans is high at around 35%, but low in animals at 2% [14–16]. Although the mortality rate in Thailand is low in most animals, the seroprevalence in pigs and elephants is higher (25%) than that of other animals (<2%), such as cattle, pigs, goats, sheep, and deer [17–19].

Most studies on melioidosis primarily focus on human and livestock cases [12, 14–19], often neglecting companion animals such as dogs [20–22], which are the most popular companion animals [23]. It should be noted, however, that dogs may develop melioidosis in contact with contaminated soil or water, especially in endemic areas, and may transmit it to their owners. Therefore, understanding the prevalence, clinical signs, and transmission of canine melioidosis are crucial for developing comprehensive control and prevention strategies.

At present, there is a very limited report of canine melioidosis worldwide, and no epidemic study has been conducted to date [20–22]. This study aimed to estimate the seroprevalence of melioidosis in sheltered dogs in southern Thailand and to determine whether the disease is associated with blood chemistry, hematology, or blood pathogen infection.

## Materials and Methods

### Ethical approval

The Institutional Animal Care and Use Committee of Walailak University (ID: 63–036) and the Institutional Biosafety Committee of Walailak University (ID: 66–046) approved this project for animal ethics and biosafety.

### Study period and location

The blood samples were collected in March 2021 at Nakhon Si Thammarat Province, Thailand. The hematology and serum biochemistry analyses were conducted in March 2021 at Animal Hospital of Walailak University. The remaining serum was separated and kept in an –80 °C freezer until melioidosis was analyzed in November 2023 at the Centre of Excellence Research for Melioidosis and Microorganisms, Walailak University.

### Animals

This shelter is the largest in southern Thailand and is located in the province of Nakhon Si Thammarat. He receives dogs from the southern region. The shelter was 2000 m^2^ and consisted of concrete with a 50% roof and a 50% soil area with several trees. Dogs were fed commercial food and water ad libitum. All dogs received annual vaccinations against rabies, distemper, adenovirus type 2, parainfluenza, parvovirus, leptospirosis, and coronavirus, as well as monthly deworming and tick prevention by injecting ivermectin, dropping the Frontline, and washing with tick shampoo. Blood was collected from the cephalic veins of 156 dogs (40% of the total population). Hematology and blood chemistry tests were performed immediately while the remaining serum was frozen and analyzed for melioidosis infection. All dogs were of a mixed breed, aged between 1 and 3 years, and they were obviously healthy. Most dogs were free from ticks and fleas; only a few (5 dogs) had 1–2 ticks.

### Hematological and serum biochemistry tests

Hematological analysis and serum biochemistry tests were performed using an automated Procyte Dx machine (IDEXX Laboratories, Maine, USA). The parameters included red blood cell count (RBC), hemoglobin, hematocrit, mean corpuscular volume, mean corpuscular hemoglobin, mean corpuscular hemoglobin concentration, RBC distribution width, total white blood cells (WBC), number of neutrophils, lymphocytes, and platelet (PLT) counts. Serum biochemistry analyses included alanine aminotransferase, aspartate aminotransferase (AST), blood urea nitrogen, and creatinine.

### Indirect hemagglutination assay (IHA)

IHA kits were purchased from the Department of Medical Science, Ministry of Public Health, Thailand. We performed the IHA technique following a previous study by Lantong *et al*. [24]. Serum samples were inactivated at 56°C for 30 min. Heterophile antibodies were absorbed in 5% unsensitized sheep RBCs (SRBCs) at oom temperature (25°C) for 1 h. Absorbed serum was serially diluted 2-fold in 96-well plates. Dilutions from 1:10 to 1:5120 were made in 0.15 M phosphate-buffered saline containing 0.5% bovine serum albumin. Fifty microliters of serum were added to each well, and then, 25 μL of 1% sensitized SRBC was added, except for the control well, which contained unsensitized SRBCs. A plate was covered with aluminum foil, gently shaken for 2 min, and incubated for 2 h at 25°C. Agglutination was observed, and the titer of the last dilution that showed more than 50% hemagglutination was recorded. A cutoff value of 1:160 was used as seropositivity or to indicate that the patient was currently or recently exposed to *B. pseudomallei* or infected. This particular threshold was chosen due to the high sensitivity and specificity of human studies [25]. This cutoff value is commonly used in humans and animals [14, 17, 26]. A titer between 1:20 and 1:80 was used to indicate the previous exposure or infection.

### Identification of bacteria from the environment

Soil samples were cultured according to a previous consensus guideline [27]. Three 250-mL samples of underground water (for feeding and cleaning) were collected for water samples. Each water sample was filtered through a 0.45-m membrane using a Whatman MBS I Microbiological Membrane Filtration System (Rocker Scientific, New Taipei City, Taiwan). Subsequently, membranes were transferred onto Ashdown’s agar plates and incubated at 37°C for 48 h. Any suspected *B. pseudomallei* colonies were collected from all three agar plates for further species confirmation by polymerase chain reaction (PCR) for detecting type three secretion system 1 gene (TTS1) [16, 28].

### Blood pathogen detection

*Babesia canis vogeli*, *Ehrlichia canis*, *Hepatozoon canis*, and *Anaplasma platys* were investigated using conventional PCR as described by Sontigun *et al*. [2]. DNA was extracted using an E.Z.N.A.^®^ Blood DNA Kit (Omega Bio-Tek, Norcross, GA, USA). The extracted DNA concentration was measured using a Nano-Drop™ spectrophotometer (ThermoFisher Scientific, MA, USA). DreamTaq Green Master Mix (2×) (ThermoScientific, Vilnius, Lithuania) was used as the PCR master mix. A Mastercycler Pro S machine (Eppendorf AG, Hamburg, Germany) was used for PCR. Genomic DNA of known blood pathogens was used as a positive control, and nuclease-free water was used as a negative control for each assay. DNA sequencing (Novogene, Singapore) confirmed the PCR products.

### Statistical analysis

Jamovi version 2.0 was used for statistical analysis [29]. Significant differences in blood test results between dogs with and without melioidosis were analyzed using an independent Student’s t-test. Fisher’s exact test with a two-tailed p-value was used to examine the potential correlation between the frequency of melioidosis and two factors: blood pathogen infection and thrombocytopenia. The effect of melioidosis and blood pathogen infection on PLT counts was examined using one-way analysis of variance and Tukey’s *post hoc* test. p < 0.05 was considered statistically significant.

## Results

### Seroprevalence of canine melioidosis

No signs of melioidosis were observed in any dog. Table-1 shows the titers of the antibodies against *B. pseudomallei*. Nine of the 156 dogs (5.77%) had a titer 1 > 1:160, indicating seropositivity for melioidosis. Twenty-one of the 156 dogs (13.46%) had a titer between 1:20 and 1:80, indicating a previous infection. One hundred and twenty-six dogs (80.77%) (titer 1:20) were negative for *B. pseudomallei* antibody, whereas *B. pseudomallei* was positive in both soil and water samples.

**Table-1:** Ab titers against *Burkholderia pseudomallei*.

Ab titer	Number of dogs	Percentage
<1:20	126	80.77
1:20	14	8.97
1:40	3	1.92
1:80	4	2.56
1:160	1	0.64
1:320	3	1.92
1:640	3	1.92
1:1280	2	1.28
Total	156	

Ab: Antibody

### Blood profile and canine melioidosis

There was no statistical difference in RBC parameters (p > 0.05) between seropositive and seronegative dogs (Table-2) [30]. The number of WBC, neutrophils, and PLTs tended to be lower but higher in lymphocytes than in uninfected dogs (Table-3) [31]. With regard to serum biochemistry, dogs with infection tended to have higher AST levels than uninfected dogs (Table-4) [31]. No significant difference (p > 0.05) was observed between dogs with and without melioidosis.

**Table-2:** RBC profile in dogs with or without melioidosis.

Group	RBC (10^6^/uL)	Hb (g/dL)	Hct (%)	MCV (fL)	MCH (pg)	MCHC (d/dL)	RDW (%)
Seronegative	5.94 ± 0.71	12.91 ± 0.24	39.96 ± 0.71	67.65 ± 0.53	21.80 ± 0.12	32.09 ± 0.28	14.64 ± 0.16
Seropositive	5.98 ± 0.10	12.73 ± 1.38	39.11 ± 4.31	66.73 ± 2.09	21.64 ± 0.60	32.49 ± 0.29	14.56 ± 1.30
Reference value [30]	4.95–7.87	11.9–18.9	35–57	66–77	19.5–24.5	32–36	12–15

The data are represented as mean ± standard error. RBC=Total red blood cell count, Hb=Hemoglobin, Hct=Hematocrit, MCV=Mean corpuscular volume, MCH=Mean corpuscular hemoglobin, MCHC=Mean corpuscular hemoglobin concentration, RDW=RBC distribution width

**Table-3:** WBC and PLTcounts.

Group	Total WBC (cells/cu.mm)	Neutrophil (cells/cu.mm)	Lymphocyte (cells/cu.mm)	PLTs (cells/cu.mm)
Seronegative	13,185.86 ± 386.70	7,797.99 ± 279.94	4,514.33 ± 255.95	106,157.89 ± 6,088.19
Seropositive	11,680.00 ± 1161.68	5,921.48 ± 715.51	5,131.44 ± 1,601.70	79,222.22 ± 18,804.20
Reference value [31]	5,000–14,000	2,900–12,000	400–2,900	200,000–500,000

The data are represented as mean ± standard error. WBC=White blood cell, PLT=Platelet

**Table-4:** Serum biochemistry in infected *Burkholderia pseudomallei* and non-infected dogs.

Group	BUN (mg/dL)	CREA (mg/dL)	AST (U/L)	ALT (U/L)
Seronegative	20.33 ± 0.79	1.34 ± 0.04	50.03 ± 2.67	47.14 ± 5.32
Seropositive	27.66 ± 5.95	1.51 ± 0.27	93.60 ± 24.55	34.00 ± 7.46
Reference value [31]	8–28	0.5–1.7	13–15	10–109

The data are represented as mean ± standard error. ALT=Alanine aminotransferase, AST=Aspartate aminotransferase, BUN=Blood urea nitrogen, CREA=Creatinine

### Correlation between blood pathogen infection and melioidosis

Infection with blood pathogens was found in 78 of 156 dogs (50.00%). The highest infection rate was observed for *E. canis* (41.67%, n = 65), followed by *B. canis vogeli* (17.95%, n = 28), *A. platys* (14.74%, n = 23), and *H. canis* (1.92%, n = 3). Furthermore, coinfections involving these blood pathogens were identified, including *A. platys* + *E. canis* at 5.77% (n = 9), *A. platys* + *B. canis vogeli* at 0.64% (n = 1), *E. canis* + *B. canis vogeli* at 7.69% (n = 12), *E. canis* + *H. canis* at 0.64% (n = 1), and *A. platys* + *E. canis* + *B. canis vogeli* at 5.13% (n = 8).

Table-5 shows the correlation between melioidosis and pathogens. Seven out of 79 dogs (8.86%) with blood pathogens were seropositive for melioidosis, whereas two out of 77 dogs (2.59%) without blood pathogens were seropositive. However, there was no statistically significant association between *B. pseudomallei* infection and blood pathogens (p > 0.05).

**Table-5:** Number of dogs infected with *Burkholderia pseudomallei* and/or blood pathogens.

Blood pathogens	Group	Melioidosis		

		Positive	Negative	Total
	Positive	6	72	78
	Negative	3	75	77
	Total	9	147	156

No significant correlation was observed (p > 0.05) between blood pathogens and melioidosis

### Correlation between thrombocytopenia and melioidosis

Among the hematological changes, PLT count reduction in seropositive dogs appeared to be lower (1.3 times) than that in seronegative dogs. Therefore, the relationship between PLT count and melioidosis was further investigated. Table-6 shows the correlation between seropositive dogs and thrombocytopenia (PLT count <200,000). All dogs with melioidosis had thrombocytopenia (n = 9/9), whereas only a minority of dogs with thrombocytopenia were infected with *B. pseudomallei* (n = 9/29, 6.97%). However, there was no statistically significant association between thrombocytopenia and melioidosis (p > 0.05).

**Table-6:** Correlation between thrombocytopenia and melioidosis.

Platelet count (cell/mm^3^)	Group	Melioidosis		

		Positive	Negative	Total
	<200000	9	120	129
	>200000	0	27	27
	Total	9	147	156

No significant correlation was observed (p > 0.05) between platelet count and melioidosis

### Effect of melioidosis and blood pathogen infection on PLT counts

Table-7 shows specific information regarding dogs diagnosed with melioidosis (antibody titer), their concurrent blood pathogen infections, and their corresponding PLT counts. Table-8 shows a comparison of PLT counts among the groups of dogs. Regarding the PLT issue, dogs that tested positive for both melioidosis and blood pathogens exhibited a significantly lower (p < 0.01) PLT count (49,167 ± 7,167) than dogs that tested positive for melioidosis but did not have blood pathogen infections (139,333 ± 29,913). In addition, dogs that tested positive for blood pathogens but not melioidosis also had a significantly lower (p < 0.01) PLT count (81,825 ± 7,980) than dogs that tested negative for both blood pathogens and melioidosis (127,411 ± 8,634). The combination of melioidosis and blood pathogen coinfection appeared to lead to a greater decrease in PLT count compared with dogs infected solely with blood pathogens; however, no statistically significant difference was observed (p > 0.05).

**Table-7:** Details of Ab titer and blood pathogens.

Dog ID	Ab titer	Blood pathogens	Platelet count (cells/mm^3^)
A1	1: 640	*E. canis*	50,000
C3	1: 640	Negative	157,000
C13	1: 320	*E. canis* and *B. canis vogeli*	84,000
C16	1: 160	*A. platys* and *E. canis*	42,000
D2	1: 320	*E. canis*	41,000
D5	1:320	*E. canis*	38,000
H12	1: 1280	Negative	81,000
H15	1: 1280	Negative	180,000
H20	1: 640	*E. canis*	40,000

*E. canis=Ehrlichia canis, B. canis vogeli=Babesia canis vogeli, A. platys=Anaplasma platys,* Ab=Antibody

**Table-8:** PLT count among the groups of dogs.

Group of dogs	n	PLT count (cells/mm^3^)
Positive melioidosis/positive blood pathogens	6	49,167 ± 7,167^a^
Positive melioidosis/negative blood pathogens	3	139,333 ± 29,913^b^
Negative melioidosis/positive blood pathogens	72	81,825 ± 7,980^a^
Negative melioidosis/negative blood pathogens	75	127,411 ± 8,634^b^

^a,b^Represents a significant difference (p < 0.01). PLT=Platelet

## Discussion

To the best of our knowledge, this is the first study on canine melioidosis infection in dogs. According to the results of this study, approximately 6% of the tested dogs showed seropositivity for *B. pseudomallei*. It is worth noting that canine melioidosis has been documented in only three articles, encompassing a total of five dogs, and two of these studies (involving two dogs) featured canine cases from Thailand [20–22]. However, PLT may play an important role in *B. pseudomallei* infection because thrombocytopenia has been previously reported in one dog with melioidosis [20] and in 31% of human patients [32, 33]. Patients with severe symptoms of melioidosis have significantly lower PLT counts than those with less severe disease [32, 33]. Using a murine melioidosis model, it has been discovered that animals with low PLT counts have a higher mortality rate and more severe disease than normal mice [33]. In our study, seropositive dogs tended to have lower PLT counts than seronegative dogs, and coinfection of melioidosis with blood pathogens could reduce PLT counts more than infection with melioidosis alone. However, melioidosis and thrombocytopenia were not significantly correlated.

Blood pathogens are commonly observed in several animal species in Thailand, including dogs [2, 34]. A reduction in PLTs is highly associated with blood pathogen infections, such as *E. canis, B. vogeli*, or *A. platys* [2, 8], and a lower number of PLTs may increase the risk of other infections [35, 36]. However, this study had no significant correlation between melioidosis and blood pathogen infection.

In the present study, we observed a trend toward elevated serum AST (also known as SGOT) levels in dogs seropositive for melioidosis. Notably, higher levels of AST have been reported in many human patients with septicemic melioidosis in Thailand [37]. It is well known that elevated AST levels are typically associated with muscle injury. Muscle infections caused by *B. pseudomallei* have been documented in humans but not in animals [38–40]. Consequently, further research is required to explore the potential relationship between canine melioidosis and muscle injury.

Anemia and leukocytosis have previously been reported in dogs with melioidosis [20] but not in our study. This difference can be attributed to the fact that the dogs in our study had asymptomatic infections, characterized by normal appetite and absence of clinical signs. In contrast, a dog in a previous report had clinical melioidosis [20]. It should be noted that most animals with melioidosis appear to be in good health [14, 16–18, 41]. In some cases, antemortem examinations conducted in slaughterhouses have revealed abscesses in visceral organs such as the liver, lung, and spleen [16].

Bacterial culture is the gold standard for detecting *B. pseudomallei* infection; however, this technique is used in cases of sepsis or lesions such as abscesses. Therefore, the IHA technique is still widely used for screening disease (epidemiology) and individual case detection because it is cheap and relatively easy to perform [42]. There are several limitations to the use of IHA for diagnosing clinical melioidosis. Approximately 73% of patients with acute culture-confirmed melioidosis are seropositive. A negative IHA titer cannot rule out the potential for latent disease because 13% of survivors had a negative IHA titer throughout the study despite having clinical signs and their specimens testing positive for bacteria culture [42]. Hence, the diagnosis of clinical melioidosis cannot rely solely on IHA; it necessitates additional diagnostic criteria, including the presence of clinical symptoms in patients and confirmation through bacterial culture from specimens such as abscesses, blood, urine, and sputum.

In addition, IHA may be the most accurate method to identify asymptomatic melioidosis because most seropositive cases in humans and animals did not manifest any clinical symptoms [16, 17, 43]. A seroprevalence study in pigs and elephants showed a high seropositivity rate (25%), but none of these animals showed any signs of melioidosis. Clinical melioidosis in certain pigs has only been confirmed by post-mortem examinations conducted in slaughterhouses [16, 17]. It is conceivable that the seropositive dogs observed in this study represent true infections in young, healthy individuals who can remain asymptomatic. Severe and acute disease, on the other hand, could result from either a high initial bacterial exposure or a compromised immune response, which appears to occur in only a small proportion of otherwise healthy adults who become infected [43].

In this study, both water and soil samples tested positive for *B. pseudomallei* bacteria. It is plausible that these dogs were exposed to these bacteria because the water was used for cleaning and feeding, and the dogs often had access to the soil that they dug. Although all dogs in the shelter had a higher risk of exposure to the bacteria, there were no cases of clinical melioidosis and only a few dogs tested seropositive. These results are consistent with previous findings that *B. pseudomallei* is commonly detected in natural water and soil in southern Thailand [13, 16, 31, 44].

## Conclusion

The melioidosis seroprevalence in dogs was low (5.77%), and all dogs showed no clinical signs. No significant correlation was observed between PLT count, blood pathogen infection, and melioidosis. However, coinfection of melioidosis with blood pathogens significantly reduces the number of PLTs compared with a single infection. To address the knowledge gap, future research should focus on conducting seroprevalence studies in the general dog population.

## Authors’ Contributions

PF: Methodology, validation, formal analysis, and visualization. NS and WB: Methodology and resources WKK: Supervision, methodology, and resource. OH: Supervision and visualization. TW: Conception, methodology, validation, formal analysis, investigation, resource, data curation, writing the original draft, visualization, project administration, and funding acquisition. All authors have read, reviewed, and edited the final manuscript.
